# Application of modified Michaelis – Menten equations for determination of enzyme inducing and inhibiting drugs

**DOI:** 10.1186/s40360-021-00521-x

**Published:** 2021-10-11

**Authors:** Saganuwan Alhaji Saganuwan

**Affiliations:** Department of Veterinary Pharmacology and Toxicology, College of Veterinary Medicine, Federal University of Agriculture, P.M.B.2373, Makurdi, Benue State Nigeria

**Keywords:** Enzymology, Drug, Michaelis-Menten equation, Pharmacokinetics, Efficacy, Toxicity

## Abstract

**Background:**

Pharmacokinetics (PK) is the process of absorption, distribution, metabolism and elimination (ADME) of drugs. Some drugs undergo zero-order kinetics (ethyl alcohol), first order kinetics (piroxicam) and mixed order kinetics (ascorbic acid). Drugs that undergo Michaelis-Menten metabolism are characterized by either increased or decreased metabolism constant (Km) and maximum velocity (Vmax) of enzyme reaction. Hence literatures were searched with a view to translating in vitro-in vivo enzyme kinetics to pharmacokinetic/pharmacodynamic parameters for determination of enzyme inducing and inhibiting drugs, in order to achieve optimal clinical efficacy and safety.

**Methods:**

A narrative review of retrospective secondary data on drugs, their metabolites, Vmax and Km, generated in the laboratory and clinical environments was adopted, using inclusion and exclusion criteria. Key word search strategy was applied, to assess databases of published articles on enzyme inducing and inhibiting drugs, that obey Michaelis-Menten kinetics. In vitro and in vivo kinetic parameters, such as concentration of substrate, rate of endogenous substrate production, cellular metabolic rate, initial velocity of metabolism, intrinsic clearance, percent saturation and unsaturation of the enzyme substrate, were calculated using original and modified formulas. Years and numbers of searched publications, types of equations and their applications were recorded.

**Results:**

A total of fifty-six formulas both established and modified were applied in the present study. Findings have shown that theophylline, voriconazole, phenytoin, thiopental, fluorouracil, thyamine and thymidine are enzyme inducers whereas, mibefradil, metronidazole, isoniazid and puromicin are enzyme inhibitors. They are metabolized and eliminated according to Michaelis-Menten principle. The order could be mixed but may change to zero or first order, depending on drug concentration, frequency and route of drug administration.

**Conclusion:**

Hence, pharmacokinetic-pharmacodynamic translation can be optimally achieved by incorporating, newly modified Michaelis-Menten equations into pharmacokinetic formulas for clinical efficacy and safety of the enzyme inducing and inhibiting therapeutic agents used in laboratory and clinical settings.

## Background

Elimination half-life, volume of distribution which is responsible for drug transport to sites of metabolism, maximum plasma concentration which could determine metabolism, enzyme saturation and elimination and maximum time (Tmax) reached, may be used to determine time of enzyme saturation, pharmacokinetic and pharmacodynamic response of drugs [1]. Cell organelles involved in metabolism and their dimensions are adiposomes (20 nm–I *μm*), amphisomes (822 ± 37 nm), apicoplast (0.15–1.5 *μm*), autophagosome (0.15–1.5 *μm*), chloroplast (2–10 *μm*), enlargesome, enosome (30–100 nm), lysosome (0.1–1.2 nm), melanosome (≃ 500 nm), mitochondria (0.5–5 *μm*), nucleus (≃ 10 *μm*), peroxisome (500 nm), phagosome (0.9–3 *μm*), secretory granule (820 ± 16 *μm*) and secretory synaptosome (0.5–3 *μm*). Hence analysis of organelle is vital for description of biochemical, molecular and physiological processes that are involved in pathogenesis of diseases, embryogeny, tissue differentiation, aging and treatment of various diseases [2]. Michaelis-Menten equation was used to estimate Km and Vmax from initial rate of reaction (V_O_), at substrate concentration (Cs). However, it was assumed that the elimination rate of drug was partly a function of the drug concentration. Hence a minor change in the initial parameter may cause a large change in the final estimates [3]. Simple model incorporating Michaelis-Menten type elimination with one compartment model, using intravenous bolus had been published [4]. Nonlinear regression algorithms with numerical integration have been used to generate pharmacokinetic parameters. Administration of two or more doses yields better translation [3]. Hence preclinical pharmacokinetic studies remove some drugs out of discovery process [5]. Therefore the aim of the study is to integrate in vitro kinetics with in vivo kinetics with a view to optimizing clinical efficacy and safety of enzyme inducing and inhibiting drugs. As such the research question is, can Michaelis-Menten equation be modified for determination of liver enzyme inducing and inhibiting drugs and xenobiotics?

## Methods

### Study design

A narrative review of retrospective secondary data on drugs and their metabolites, Vmax and Km, generated in the laboratory and clinical settings was adopted. The literature searched was carried out at Mannex Business Computer Centre, High Level Makurdi and at Crystalporttech Computer Centre Minna all in Nigeria between 1st July, 2019 and 30th June, 2020. Manuscripts published by Elsevier, Sage, Springer, Springer Nature, Tailor and Francis, Wiley among others were searched using Google Scholar, PubMed/Medline, Cochrane among others. The titles of the manuscripts were cross-checked with their contents and the fields of authors’ research. A total of one hundred and twenty-one papers published between 1963 and 2020 were examined. Keywords search strategy was adopted, to access databases of the published articles, on drugs that obey Michaelis-Menten principle (Table 1). Literature search entitled “pharmacokinetics of drugs that obey Michaclis-Menten principle of metabolism” was carried out with intent to identifying equations that could be used in identification of drugs that obey zero-order, first order and mixed-order kinetics. Keywords such as pharmacokinetic, metabolism, elimination, enzyme substrate, enzyme kinetic, inducer, inhibitor, metabolite, drug-combination, kinetic modeling, in vitro, in vivo among others were used to search for drugs that obey Michaelis-Menten principle. The inclusion criteria were, the papers published in English Languages, drugs whose metabolism constant (Km) and maximum velocity (Vmax) have been reported, and the drugs that were either enzyme inducing or inhibiting, whose metabolic processes resulted in production of metabolites at specific doses, using different routes of administration. In vitro and in vivo equations have been included. The exclusion criteria were drugs that have no clinical application, and whose metabolic processes do not obey Michaelis-Menten principles. All articles published before 1963 on Michaelis-Menten principle have been excluded also. Maximum velocity of metabolism, metabolic rate constant, quantities of substrate and factors associated with metabolic processes of the drugs were also determined. Data generated from modified Michaclis-Menten equations (Table 2) were translated to kinetic parameters that were in turn guarded by metabolic processes of the drugs [6–51].

**Table 1:** Types of searched articles, years of search and numbers of the articles sampled

Article Type	Year of Publication	Number of Publication	Search Terms	Remarks
Thesis	2008	1	Kinetic modelling	Integration of kinetic equations
Chapter	2015 and 2019	2	Kinetics and dynamics of antimicrobials	Metronidazole, Isoniazid, Tylosin, Mibefradil
Conference Proceeding	2009	1	Drugs that obey Michaelis - Menten principle	Voriconazole
Journals	1963–2020	112	Drugs that obey mixed, first and zero order kinetics; metabolism; elimination; enzyme substrate; inhibitor; inducer; metabolite; in vitro; in vivo	Kinetic parameters of eleven enzymes; (4) inhibiting and (7) inducing drugs were obtained.
Book	1975–2015	5	Enzymatic kinetics	Types of orders of kinetics obtained

**Table 2:** Statistics of in vivo kinetic equations

Equation Types	Number of Equations	Modified Equations	Applications	Remarks
Michaelis – Menten	1	3	Determination of enzyme saturation	Not perfect
Non – linear kinetic	17	17	Determination of rate – limiting and non – rate limiting enzymes in mixed order kinetics	Derived from Michaelis – Menten equation
Cellular metabolic rate	5	3	Determines minimum and maximum level of metabolism in cells	Obeys Eadie-Hofstee plot
Kinetics beyond Michaelis – Menten	11	6	Determination of drugs metabolized by allosteric enzymes	Benzodiazepines as the examples
Non – linear mixed kinetic	15	10	Determination of in vivo kinetics	Simple method of determining drug disposition
Narrow therapeutic window	2	0	Determination of safe doses	It is good for renal impaired patient
Combined Michaelis – Menten	3	0	Identification of liver enzyme inducers/inhibitors	In vitro/In vivo integrated for balanced kinetics/dynamics

### Integration of in vitro-in vivo kinetic equations

#### Michaelis-Menten and related equations

Michaelis – Menten equations are given below

1 $$ {\mathrm{V}}_0=\mathrm{Vm}\times \mathrm{C}/\mathrm{Km}+\mathrm{C}\ \left(\mathrm{Classic}\kern0.17em \mathrm{hyperbolic}\kern0.17em \mathrm{plot}\right) $$

2 $$ \frac{1}{k_o}=\frac{1}{{\mathrm{V}}_m}+\left[\frac{km}{0.632{\mathrm{V}}_m}\right]\frac{1}{{\mathrm{C}}_o}\left(\mathrm{Modified}\kern0.17em \mathrm{Line}\hbox{-} \mathrm{Weaver}\hbox{-} \mathrm{Burk}\ \mathrm{plot}\right) $$

3 $$ \mathrm{Percent}\kern0.17em \mathrm{saturation}\left(\%\right)=\frac{100\mathrm{C}}{km+\mathrm{C}} $$

Vo = Initial velocity of reaction; Vmax = Maximal velocity of reaction; C=Concentration of enzyme substrate; Km = Metabolism constant; Ko = Initial metabolism constant

Equations 1–3 can be used for determination of enzyme saturation during enzyme induction and inhibition process.

#### Derived equations for calculation of non-linear drug kinetic parameters

Substitute clearance (Cl) for V in the eq. 1.

4 $$ \mathrm{C}l=\frac{{\mathrm{V}}_{max}\mathrm{C}}{km+\mathrm{C}} $$

Also substitute V for DR (dose rate) and C for Css (steady-state concentration in eq. (1).

5 $$ DR=\frac{{\mathrm{V}}_{max}\mathrm{Css}}{km+\mathrm{Css}} $$

Also the modified Michaelis-Menten equation for consumed substrate that is endogenously produced is presented as

6 $$ V=\frac{{\mathrm{V}}_{max}\times S}{km+S}+R $$

Where R is the rate of endogenous substrate production

7 $$ \mathrm{Vo}\kern0.30em \times \kern0.30em \mathrm{Clint}=\mathrm{R} $$

8 $$ Clearance\;(Cl)=\raisebox{1ex}{$ DR$}\!\left/ \!\raisebox{-1ex}{${C}_{SS}$}\right. $$

The eq. 5 and 7 are related.

Therefore

9 $$ DR= Cl\times Css $$

Equate equations,4, 5 and 8

10 $$ DR=\frac{V_{max}\times {C}_{SS}}{km+{C}_{SS}}= Cl\times {C}_{SS} $$

11 $$ Cl=\frac{V_{max}\times {C}_{SS}}{km+{C}_{SS}}\times \frac{1}{C_{SS}} $$

Hence

12 $$ \mathrm{Css}\times \mathrm{Clu}=\mathrm{Urinary}\ \mathrm{excretion} $$

13 $$ {k}_o= Vmax+ urinary\ excretion $$

At low rate of infusion when

14 $$ \mathrm{Css}<<\mathrm{km},{\mathrm{K}}_{\mathrm{o}}= Css\left( Clu+V\mathit{\max}/ Km\right) $$

15 $$ \mathrm{Loading}\kern0.17em \mathrm{dose}\;\left(\mathrm{LD}\right)= Css\times Vd $$

16 $$ \mathrm{Enzyme}\kern0.17em \mathrm{inhibition}\kern0.17em \mathrm{constant}\ \left({k}_i\right)=0.05\times 1{C}_{50} $$

The maximal in vitro inhibitory concentration (I_*max*_) is the *C*_*max*_ in human plasma

Therefore

17 $$ \mathrm{in}\ \mathrm{vitro}\ \mathrm{in}\mathrm{hibitory}\ \mathrm{potential}\ \left({\mathrm{I}}_P\right)={\mathrm{I}}_{max}/{k}_i $$

It translates to

18 $$ \mathrm{in}\ \mathrm{vivo}\ \mathrm{in}\mathrm{hibitory}\ \mathrm{potential}={\mathrm{C}}_{\mathrm{max}}/\mathrm{ki} $$

Whereas ki is the plasma inhibitory constant

Equation (17) is identical with AUC ratio

19 $$ \mathrm{Efflux}\ \mathrm{ratio}\ \left(\mathrm{Er}\right)\ \mathrm{for}\ \mathrm{drug}\ \mathrm{moving}\ \mathrm{across}\ \mathrm{barrier}=\mathrm{Km}/\mathrm{Ki}\ \mathrm{or}\ \mathrm{Km}/{\mathrm{IC}}_{50} $$

20 $$ \mathrm{The}\ \mathrm{carrier}-\mathrm{mediated}\ \mathrm{permeability}\ \left(\mathrm{Pm}\right)=\mathrm{Jmax}/\mathrm{Km}+\mathrm{Co} $$

Clint = Intrinsic clearance; S=Substrate; Css- Steady-state concentration of drug;Clu-Urinary clearance; Vd = Volume of distribution; Inhibitory concentration 50; Jmax = Maximal carrier-mediated flux; Co = Initial donor concentration of the substance; Km = Metabolism constant [16]. At Css where metabolism is saturated, enzyme velocity approaches Vmax [49]. Therefore eqs. 4–20 can be used to determine rate-limiting and non-rate limiting enzymes that participate in zero order-first order kinetics (mixed order kinetics).

#### Equations for calculation of drug metabolic rate in cell

21 $$ Metabolic\ rate\ (MR)={aM}^{3/4} $$

a = Constant for all mammals; M = Body mass in kg; metabolic rate is expressed in moles of oxygen consumed /second. But cell metabolic rate is:

22 $$ \left(\mathrm{CMR}\right)=\frac{MR}{Number\ of\ cells}={aM}^{-1/4} $$

However, oxygen consumption rate per cell increases as body mass decreases.

23 $$ \left(\mathrm{CMR}\right)=\frac{V_{max}}{P}\left(\mathrm{obeys}\ \mathrm{Eadie}-\mathrm{Hofstee}\ \mathrm{plot}\right) $$

P = 2.3026 [52].

24 $$ but\ \frac{ds}{dt}=\frac{-{V}_{max}\ S}{km+S}=-\mu (s) $$

25 $$ Volume\ of\ drug\ (V)=\frac{Quantity\ of\ drug}{Concentration} $$

Equation 21 is referred to as specific growth function in cell growth modeling [53].

$$ {V}_{max}\left( mg/ hr\right); km\ \left( mg/L\right) $$

Equations 21–25 can be used to determine minimum and maximum level of drug metabolism in individual cells, which is a function of Eadie-Hofstee plot. At maximum velocity of enzyme reaction, the metabolism constant becomes negative. Hence removal of the drug from body system is invariably delayed.

#### Kinetic equations beyond Michaelis-Menten order

For reaction beyond Michaelis-Menten order, the following equation can be used. The equation describes the dependence of enzyme-catalyzed reaction on the concentration of substrate using catalytic constant (*K*_*cat*_) and Michaelis-Menten constant (km).The *K*_*cat*_ determines the maximum rate of the reaction at saturating substrate concentration *V*_*max*_.

Therefore

26 $$ {V}_{max}={K}_{cat}\times {E}_T $$

$$ 1\ {\mathsf{K}}_{\mathsf{cat}}=60\ \mathrm{mol}/\min =6\ \mathrm{x}\ {\mathsf{10}}^{\mathsf{7}}\mathrm{unit}\mathrm{s};1\ \mathrm{unit}=1\upmu \mathrm{mol}/\min =16.67\ \mathrm{nkat} $$ [54].

Where *E*_*T*_= the total enzyme concentration; km = the substrate concentration at which reaction is half of *V*_*max*_ [9].

27 $$ \mathrm{Enzyme}\ \mathrm{substrate}(ES)=\frac{Kinfus}{K_{cat}} $$

28 $$ When\ S\ll km\  cleareance\ (Cl)=\frac{V_{max}}{km}\ \left(\mathrm{Linear}\ \mathrm{Eadie}-\mathrm{Hofstee}\ \mathrm{plot}\right) $$

Note that the enzyme will never reach its full activation.

When

29 $$ \mathrm{S}>>\mathrm{km},\mathrm{clearance}={V}_{max}\left[S\right] $$

Note that the reaction is at full speed.

For acute dosing Clearance

30 $$ (Cl)=\frac{V_{max}}{km+S} $$

31 $$ \mathrm{Rate}= Cl\ X\ S=\frac{vmax}{km+s} XS $$

32 $$ {ES}_{SS}=\frac{Kinfus}{k_{cat}} $$

33 $$ A=\frac{v}{k} $$

Where A is a lower boundary for concentration, C(t)

Upper boundary for C(t) is

34 $$ A=\frac{v}{k}+B $$

*B*= Maximum concentration C(t).

Replace $\frac{V}{K\ }$ by KE in the eqs. 33 and 34

Hence

35 $$ A= KE $$

36 $$ A= KE+B $$

The standard kinetic parameters for intravenously administered drugs are Co (2.0), V (0.22) and K (0.11). Whereas KA (1.5), V (2.05), K (5.0), D_1_ (10.0), D_2_ (20.0) and D_3_ (40.0) have been reported for first order kinetics [3]. Equations 26–36 can be applied for determination of drugs that are metabolized by allosteric enzymes which have many active sites that are highly cooperative. The affinity of one active site can be affected by drug binding to another active site. Examples of such drugs are benzodiazepines (diazepam, lorazepam) that bind to ionotropic gama aminobutyric acid receptor. The drugs are either positive or negative Guanosine-protein coupled receptor. Hence they are nonconvalent.

#### Non-compartmental/non-linear mixed kinetic equations

37 $$ (Cl)= Vd\times \beta $$

38 $$ (Cl)=\frac{Vd}{MRT} $$

39 $$ Vd\times \beta =\frac{Vd}{MRT} $$

40 $$ MRT=\frac{Vd\times \beta }{Vd}=\beta $$

41 $$ AUC=\frac{Dose}{Cl} $$

42 $$ MRT=\frac{AUMC}{AUC} $$

Equate eq. (40) with (42)

43 $$ MRT=\frac{AUMC}{AUC}=\beta $$

44 $$ \beta =\frac{AUMC}{AUC} $$

45 $$ Cl=\frac{F(Dose)}{AUC} $$

46 $$ \mathrm{Terminal}\ \mathrm{half}-\mathrm{life}\ \left(T\frac{1}{2\ }\ \beta \right)=\frac{Vd}{Pcl}\times 0.693 $$

47 $$ \mathrm{Rate}\ \mathrm{constant}\ \mathrm{of}\ \mathrm{elimination}\ \left(\mathrm{K}10\right)=\frac{Cl}{Vc} $$

V_C_ = volume of central compartiment, Vd = volume of distribution; $T\frac{1}{2}\beta =$ elimination half-life, *β*= elimination rate constant; MRT = mean residence time; *AUC*= area under curve; *AUMC*= area under moment curve.

Change in concentration over time is equal to dose of drug divided by apparent volume of distribution of the drug [55].

48 $$ \frac{dc(t)}{dt}=-\frac{Vmax\ C(t)}{Km+C(t)}={C}_o=\frac{D}{V_{app}} $$

Co = Initial concentration; D=Dose of drug; Vapp = Apparent volume of distribution

49 $$ \mathrm{Vss}= Cl\times MRT=\frac{Dose_{iv}\times AUMC}{AUC} $$

50 $$ \mathrm{Rate}\ \mathrm{of}\ \mathrm{metabolism}\ \left(\mathrm{Vo}\right)= Clint\times Cs $$

51 $$ \mathrm{Clint}={V}_o/ Cs= Vmax/ Km $$

Clint = proportionality constant between rate of metabolism and the drug substrate concentration at the enzyme site (Cs) [5]. Eqs. 37–51 can be used to calculate pharmacokinetic parameters of a drug concentration in vivo over a period of time. It is a linear, quick and simple method of evaluating drug disposition.

#### Equations for adjusting serum concentration of enzyme inducing drugs

Many enzyme inducing drugs for example phenytoin, have narrow therapeutic window phenomeno. Hence their serum concentrations can be calculated for patient with good renal function as follows:

52 $$ \mathrm{Corrected}\ \mathrm{Concentration}=\frac{\mathrm{Observed}\ \mathrm{Concentration}}{0.2\ \mathrm{x}\ \mathrm{Albumin}\ \left[\mathrm{g}/\mathrm{dL}\right]+0.1} $$

The concentrations of enzyme inducing drugs for patients with end-stage renal failure are calculated thus:

53 $$ \mathrm{Corrected}\ \mathrm{Concentration}=\frac{\mathrm{Observed}\ \mathrm{Concentration}}{0.1\ \mathrm{x}\ \mathrm{Albumin}\ \left[\mathrm{g}/\mathrm{dL}\right]+0.1} $$

Equations 52 and 53 can be used to adjust therapeutic dose of enzyme inducing drugs in patients with renal impairment or failure.

#### Combination of plots of Michaelis-Menten equations

Lineweaver-Burk double reciprocal equation is given as follows:

54 $$ \frac{1}{{\mathrm{V}}_{\mathrm{o}}}=\frac{\mathrm{Km}}{{\mathrm{V}}_{\mathrm{max}}}\mathrm{x}\frac{1}{\left[\mathrm{S}\right]}+\frac{1}{{\mathrm{V}}_{\mathrm{max}}} $$

Eadie-Hofstee plot is given as follows:

55 $$ {\mathrm{V}}_{\mathrm{o}}=-\mathrm{Km}\ \mathrm{x}\ \frac{{\mathrm{V}}_{\mathrm{max}}}{\left[\mathrm{S}\right]}+{\mathrm{V}}_{\mathrm{max}} $$

Hanes-Woolf plot is given as follows:

56 $$ \frac{\left[\mathrm{S}\right]}{{\mathrm{V}}_{\mathrm{o}}}=\frac{1}{{\mathrm{V}}_{\mathrm{max}}}\left[\mathrm{S}\right]+\frac{\mathrm{Km}}{{\mathrm{V}}_{\mathrm{max}}} $$

Note that eqs. 54–56 are modifications from Michaelis-Menten eq. I understand that none of the eq. 1–56 is perfect. Hence the equations integrated herein can be for identification of liver enzyme inducing and inhibiting drugs.

Note that all the formulas 1–56 in the present context can be used in calculation of in vitro –in vivo pharmacokinetic, pharmacodynamic, and Michaelis-Menten parameters that can identify enzyme inducing and inhibiting drugs or toxicants.

### Statistical analysis

Metabolic parameters were calculated quantitatively, whereas percent saturation was calculated qualitatively and the data generated were compared. The judgement of enzyme induction and inhibition was based on increased and decreased values of measured parameters. Also data generated were presented in average ± standared error of mean (SEM). One-way analysis of variance (ANOVA) was used to analyze data, and honestly significant difference (HSD) was used to compare differences in variances at 5% level of significance [56].

## Results

Kinetic integrated eqs. (1–56) presented above could be used for calculation of pharmacokinetic/pharmacodynamic parameters, to optimize clinical efficacy and safety of drugs. The equations could also be used for assessment of toxicokinetic/toxicodynamic parameters, for identification of potential hazards. Few parameters calculated using eqs. 1, 3, 6, 7, 21 and 49 are presented in Table 3. Values of kinetic parameters were significantly lower (*p* < 0.05) among inducers as compared to inhibitors (Tables 3 & 4). Enzyme inducers were theophylline, voriconazole, phenytoin, thiopental, thymidine, thymine, fluorouracil and tylosin. Enzyme inhibitors were mibefradil, metronidazole, isoniazid and puromycin. However, zero value of puromycin substrate concentration translated to zero rate of endogenous substrate production, initial velocity and 0 % saturation. Rate of endogenous substrate production for metronidazole, phenytoin, thymidine and thymine was zero, in spite of available substrate, except for puromycin. Rate of endogenous substrate production and initial velocity of phenytoin that produced phenylhydantoin was zero, unlike phenytoin that produced hydroxyphenytoin, suggesting the later may be highly reactive. Nevertheless, the rate of endogenous substrate production of thiopental was negative, suggesting very low initial velocity of the metabolic process. In spite of zero level of rate of endogenous substrate production for thymine and thymidine, their substrate concentrations were yet high, suggesting lack of relationship between cellular metabolic rate and substrate concentration. Vmax, Cs, R, CMR, Vo, Clint, percent enzyme saturation and S/Km were low among enzyme inducers whereas Km and percent enzyme unsaturation were much higher among inducers (Tables 3 & 4).

**Table 3:** Metabolic parameters of some drugs generated from Vmax and Km using modified Michaelis-Menten equations

Drug	Binding Site	Metabolites	Dose (mg)	*V*_*max*_	Km	Cs	R	*CMR*	Vo	Clint	% saturation	S/Km	Status	pH	References
Theophylline (mg)	Adenosine receptor/RNA	1, 3 – DMU	300	34.2	14.2	1.08	0.18	14.85	2.60	2.41	7.07	0.08	Inducer	Weak base	[25]
		1 – MU	300	13.1	9.3	1.12	0.17	5.69	1.58	1.41	10.74	0.12	Inducer	Week base	[24]
		3 - MX	300	4.9	2.0	2.0	2.45	2.13	4.90	2.45	50.00	1.00	Inducer	Weak base	[25]
Mibefradil (mg)	Globulin	Dealkylated	100	6.79	70.2	1.02	0.003	2.95	0.10	0.097	1.43	0.01	Inhibitor	Base	[57]
Voriconazole (ng)	Albumin/globulin	Hydroxylated	400	114	1.15	7.67	661.2	49.51	160.33	99.13	86.96	6.67	Inducer	Acidic	[6]
Metronidazole (*μm*)	Plasma protein	2-hydroxy	300	0.366	0.911	1.00	0.00	0.16	4.02	4.02	52.32	1.10	Inhibitor	Acidic	[23]
		metronidazole	300	0.527	0.235	0.31	−2.10	0.23	0.14	2.24	56.88	1.32	Inhibitor	Acidic	[1]
Isoniazid (mg)	Protein	Isonicotinic acid	250	30.87	1.669	2.50	27.75	13.41	46.25	18.50	59.97	1.50	Inhibitor	Base	[35]
Puromicin(mg)	Ribosome	5′-monophosphate	100	193.9	0.0435	0.00	0.00	84.21	0.00	0.875	0.00	0.00	Inhibitor	Acidic-basic	[15]
Phenytoin (mg)	Na + receptor	Phenylhydantoin	300	8.25	9.43	0.049	0.00	3.58	0.00	0.043	0.52	0.01	Inducer	Base	[7]
R – Thiopental(mg)	GABAA	–	120	0.86	20.0	1.03	−0.002	0.37	0.04	0.042	4.90	0.05	Inducer	Base	[10]
Phenytoin (mg)	Na + receptor	Hydroxyphenytoin	300	28.0	91.0	1.01	0.23	12.16	0.31	0.308	1.10	0.01	Inducer	Base	[37]
S – Thiopental (mg)	GABAA	–	300	1.01	24.0	1.04	0.002	0.44	0.04	0.042	4.15	0.04	Inducer	Base	[33]
Thymidine(mg)	Nucleoside	Baminoisobutyric acid	–	2.45	45.5	1.00	0.00	1.06	0.053	0.053	2.15	0.02	Inducer	Acidic	[11]
Thymine(mg)	DNA	–	–	2.55	82.5	0.98	0.00	1.11	0.03	0.030	1.17	0.01	Inducer	Base	[12]
Fluorouracil(mg)	Albumin	Dihydrofluorouracil	–	2.16	39.8	1.02	0.006	0.94	0.06	0.054	2.50	0.03	Inducer	Base	[13]
Tylosin(mg)	Ribosome	Relomycin	110	0.018	0.37	0.58	−0.021	0.01	0.028	0.049	61.05	1.57	Inducer	Acid-basic	[1]

Keys: *Vmax* Maximal velocity (mg/h), *Km* Metabolism constant (mg/L), *Cs* Concentration of substrate (μg; ng; μm; mg), *R* Rate of endogenous substrate production (h), *CMR* Cellular metabolic rate (/h), *Vo* Initial velocity (mg/h), *Clint* Proportionality constant between rate of metabolism and the drug substrate concentration at the enzyme site Cs, *% saturation* Extent of enzyme saturation; 3-MX: 3-methylxanthine; 1,3-DMU:1,3 dimethyluric acid;1-MU:1-methyluric acid

**Table 4:** Calculated comparative Michaelis – Menten parameters of some enzyme inducing and inhibiting drugs

Drug	Vmax	Km	Cs	R	CMR	Vo	CLimt	% Saturation	% Unsaturation	S/Km
Theophylline*	17.4 ± 8.7	8.5 ± 3.5	1.4 ± 0.3	0.9 ± 0.8	7.6 ± 3.8	3.0 ± 1.0	2.1 ± 0.3	22.6 ± 0.6	77.4 ± 13.7	0.4 ± 0.3
Mibefradil**	6.8 ± 0.0^b^	70.2 ± 0.0^a^	1.0 ± 0.0^b^	0.0 ± 0.0^b^	30.0 ± 0.0^b^	0.0 ± 0.0^b^	0.1 ± 0.0^b^	1.4 ± 0.0^b^	98.6 ± 0.0^a^	0.01 ± 0.0^b^
Voriconazile*	114.0 ± 0.0^a^	1.2 ± 0.0^b^	7.7 ± 0.0^a^	661.2 ± 0.0^a^	49.5 ± 0.0^a^	160 ± 0.0^a^	99.1 ± 0.0^a^	87.0 ± 0.0^a^	13.0 ± 0.0^b^	6.7 ± 0.0^a^
Metronidazole**	0.45 ± 0.1^b^	0.6 ± 0.3^b^	0.7 ± 0.3^b^	−1.1 ± 1.1^b^	0.2 ± 0.0^b^	2.1 ± 1.9	3.1 ± 0.9^a^	54.6 ± 2.3^a^	45.4 ± 1.9^b^	1.2 ± 0.1^a^
Isoniazid**	30.9 ± 0.0^a^	1.70 ± 0.0^b^	2.5 ± 0.0^a^	27.8 ± 0.0^a^	13.4 ± 0.0^a^	46.3 ± 0.0^a^	18.5 ± 0.0^b^	60.0 ± 0.0^a^	40.0 ± 0.0^b^	1.5 ± 0.0^a^
Puromicin**	193.9 ± 0.0^a^	0.04 ± 0.0^b^	0.0 ± 0.0^b^	0.0 ± 0.0^b^	84.2 ± 0.0^a^	0.0 ± 0.0^b^	0.9 ± 0.0^b^	0.0 ± 0.0^b^	100 ± 0.0^a^	0.0 ± 0.0^b^
Phenytoin*	18.1 ± 9.9^a^	50.2 ± 40.8^a^	0.5 ± 0.5^b^	0.1 ± 0.1^b^	7.9 ± 4.3	0.2 ± 0.0^b^	0.2 ± 0.1^b^	0.8 ± 0.3^b^	99.2 ± 0.3^a^	0.01 ± 0.0^b^
Thiopental*	0.9 ± 0.1^b^	22.0 ± 2.0^a^	1.0 ± 0.0^b^	0.0 ± 0.0^b^	0.4 ± 0.0^b^	0.01 ± 0.0^b^	0.04 ± 0.0^b^	4.5 ± 0.4^b^	95.5 ± 0.4^a^	0.05 ± 0.0^b^
Thymidine*	2.5 ± 0.0^b^	45.5 ± 0.0^a^	1.0 ± 0.0^b^	0.0 ± 0.0^b^	1.1 ± 0.0^b^	0.1 ± 0.0^b^	0.1 ± 0.0^b^	2.1 ± 0.0^b^	97.9 ± 0.0^a^	0.02 ± 0.0^b^
Thymine*	2.6 ± 0.0^b^	82.5 ± 0.0^a^	1.0 ± 0.0^b^	0.0 ± 0.0^b^	1.1 ± 0.0^b^	0.03 ± 0.0^b^	0.03 ± 0.0^b^	1.2 ± 0.0^b^	98.8 ± 0.0^a^	0.01 ± 0.0^b^
Fluorouracil*	2.2 ± 0.0^b^	39.8 ± 0.0^a^	1.0 ± 0.0^b^	0.0 ± 0.0^b^	0.9 ± 0.0^b^	0.1 ± 0.0^b^	0.1 ± 0.0^b^	2.5 ± 0.0^b^	97.5 ± 0.0^a^	0.03 ± 0.0^b^
Tylosin*	0.02 ± 0.0^b^	0.4 ± 0.0^b^	0.6 ± 0.0^b^	0.01 ± 0.0^b^	0.01 ± 0.0	0.03 ± 0.0	0.05 ± 0.0	61.1 ± 0.0	39.0 ± 0.0^b^	1.6 ± 0.0^a^

Keys: * = Enzyme inducer; ** = Enzyme inhibitor; a = significantly higher (*P* < 0.05); b = significantly lower (*p* < 0.05); *Vmax* Maximal velocity (mg/h), *Km* Metabolism constant (mg/L), *Cs* Concentration of substrate (μg; ng; μm; mg), *R* Rate of endogenous substrate production (h), *CMR* Cellular metabolic rate (/h), *Vo* Initial velocity (mg/h), *Clint* Proportionality constant between rate of metabolism and the drug substrate concentration at the enzyme site Cs, *% saturation* Extent of enzyme saturation and unsaturation

## Discussion

### Non-linear application of Michaelis-Menten equation

Metabolic parameters of some drugs generated from Vmax and Km using Michaelis-Menten modified equations presented in Table 3, agree with the report indicating that nonlinear parameters, Km and Vmax obtained from steady state concentration measurements could be used to achieve optimal dosage regimen [58]. Simple intravenous, multi-dose bolus and constant injections can be described by lambert function, that fit the Michaelis-Menten parameters in designing dosing regimen, that maintains steady state plasma concentrations [55]. It is vital to maintain a concentration above minimum therapeutic level, all the times without exceeding the minimum toxic concentrations. Hence, one-compartment model with therapeutic window is relevant. Recently, one or two compartment models have been used to fit Michaelis-Menten parameters for single or multiple response data [3]. When fitting a model to Pk data for biologics with memberane bound targets, Michaelis-Menten is enough to describe the data, because only an upper bound for the receptor density can be identified [59]. If a metabolite is formed by Michaelis-Menten kinetics, linear plots of cumulative metabolite excreted in urine over time is not expected. The plasma clearance changes with dose of drug and it is expected to be different depending on dosage forms. Hence linear pharmacokinetic parameters could be evaluated using Michaelis-Menten equation [4] which states that a first order kinetics is observed at low substrate concentration, and the rate is independent of high substrate concentration [60].

### Enzyme- drug metabolite relationship

During enzyme reaction, high metabolite is formed and the enzyme suddenly becomes saturated as the substrate concentration is increased. This is observed for theophylline, voriconazole, metronidazole, isoniazid and tylosin (Tables 3 & 4). When metabolite is formed, rate of urinary excretion is equal to rate due to glomerular filtration plus rate due to active tubular secretion minus rate due to tubular re-absorption [4]. The calculated 1.43% enzyme saturation of mibefradil is corroborated by the report, indicating that metabolites of mibefradil represent 50–80% of the circulating drugs after single oral drug administration. The metabolites are formed from cytochrome P450-mediated oxidation at saturation, dealkylation and hydrolysis of the ester side chain at unsaturation [61]. Mibefradil causes life-threatening interaction with beta blockers, digoxin, verapamil, and diltiazem with consequence of developing abnormal depolarization-repolarization of the heart ventricle (QT) prolongation [3]. However, a mibefradil metabolite is a potent blocker of L-type C^2+^ current in pancreatic beta cells, which is time-dependent and poorly reversible [57]. Metabolism of voriconazole via hydroxylation is faster than via N-oxidation which are influenced by the cytochrome P450 (CYP) subfamily 2C19 genotype [39]. About 87% enzyme saturation of voriconazole in the present study connotes high level of metabolism and fast elimination. The finding is corroborated by the report indicating that voriconazole is absorbed in 2 h after oral administration, 90% bioavailable, with capacity-limited elimination, extensively distributed, 60% plasma protein bound and independent of plasma concentration. The elimination half-life is 6 h and 80% of the total dose is recovered in the urine as metabolite [42]. However, CYP2C19 and 219 genotypes are not major determinants of voriconazole metabolism [51] and 2% is excreted unchanged in urine [62]. However, metabolism of voriconazole could be autoaccelerated and controlled by cimetidine [30]. Metabolism of N-oxide voriconazole differs pre and post treatment [6].The terminal half-life is relevant to multiple dosing regimens as it controls degree of drug accumulation, concentration, fluctuations and time taken to reach equilibrium. When the process of absorption is a limiting factor, the terminal half-life reflects the extent of absorption and not the elimination process (Flip-Flop Mechanism) [63]. Isoniazid is converted to acetylisoniazid, isonicotinic acid, isonicotinylglycine, monoacetylhydrazine and diacetylhydrazine via acetylation. Fast acetylators acetylate isoniazid faster than slow acetylators 5–6 times more. Acid-labile hydrazones are also formed [64]. The formations of metabolite are via host activation of isoniazid and formation of isoniazid-NAD+ adduct [27]. Isonicotinic acid and isonicotinyl glycine are the only derivatives that contribute to isonicotinic fraction of isoniazid metabolites [35]. Therefore, toxic metabolites of isoniazid are increased in patients who are slow acetylators [65], and may account for about the 60% enzyme saturation reported in the present study. Acetyl isoniazid and diacetyl hydrazine could be determined after hydrolysis to isoniazid, acetyl hydrazine respectively [44]. Aminonucleose of puromycin is broken down to 5′-monophosphate of the nucleoside, seen 90 min after intravenous administration of puromycin [20], and could increase plasma free amino acid [15], perhaps accounting for 0 % saturation. Phenytoin is metabolized to hydroxyphenytoin and phenylidantoin which has S and R isomers [43]. When the reaction is catalyzed by CYP2C9, formation of S isomer is favoured [7] as shown by low enzyme saturation, high Vmax and high Km. However, 20–30% difference between R- and S- isomer of thiopental clearance and Vss, could account for difference in their metabolic processes, [33] as observed by differences in their reported metabolic parameters in the present study.

### Non-linearity of drug kinetics depends on Vmax and km

A low Km observed for theophylline (2 mg/L), voriconazole (1.15 mg/ L), metronidazole (0.235 mg /L), isoniazid (1.669 mg/L), puromycin (0.0345 mg/ L) and tylosin (0.37 mg /L) indicates high binding affinity as the reaction approaches Vmax rapidly. High Km observed for mibefradil (70.2 mg /L), thiopental (20.0 mg/L), theophylline (14.2 mg /L), phenytoin (91.0 mg/L), thymidine (45.5 mg/L), thymine (5-methyl uracil) (82.5 mg/L) and fluorouracil (39.8 mg/L) indicates inefficient binding of enzyme with substrate, hence V_max_ is reached when the substrate concentration is high enough to saturate the enzyme. However, S/Km ratio of 0.01–1.6 (Table 4) in the present study disagrees with the reported value of 0.01–1.0. When S < <Km, the enzymatic rate is much less than Kcat, because most of the active sites are unoccupied. Therefore, when S is low, the concentration is almost negligible, resulting to Km> > S. I_*max*_/*k*_*i*_ Values of isoniazid were identical with the AUC ratio, whereas clofazimine showed high I_*max*_/*k*_*i*_ values by four folds. Hence drugs metabolized by CYP3A4 should be carefully administered with clofazimine [66]. Hence nonlinearity is observed in metabolism involving Michaelis-Menten kinetics called saturable metabolism or mixed order kinetics. Nonlinearity may be at different levels of absorption, distribution, metabolism and excretion [67]. The pharmacokinetics with absorption and elimination in the Laplace domain could be inverted. Right skew and maxima were seen in dimensionless concentration with time plot. The tendencies of individuals to show nonlinearity in theophylline kinetics depend partly on km and *V*_*max*_ values of their respective metabolic pathway and serum theophylline concentration [24]. Age and weight have been identified to affect pharmacokinetic variability of voriconazole [32]. Chemical and antibody inhibitors have no or little effect on metronidazole 2- hydroxylation, making CYP2A6 responsible for 2- hydroxylation of metronidazole both in vitro and in vivo [68]. The general disposition of a drug is the same of local disposition at various sites in the body, whereas the local disposition is the sum of micro-disposition in the cells. Therefore, understanding of the disposition in vitro and in loci is indispensable in clinical situations [69]. Botts- Morales theory on catalytic properties of an enzyme is related to allosteric effects [70]. Hence combination of either classic-hyperbolic Eadie-Hofstee or Line-Weaver-Burk double reciprocal-Eadie-Hofstee or Hanes-Woolf-Eadie-Hofstee plot [71] may fit best for metabolism of enzyme inducing or inhibiting drugs. The negativity of S/Km and Km of Eadie-Hofstee and Hanes-Woolf plots shows that, the reactants are being consumed on the reaction. However, before products are formed contrary to the classic hyperbolic plot that shows both reactants and products are present in the reaction.

### Enzyme-drug concentration determines the rate of reaction

Subentical damped oscillations arise when Krebs cycle kinetics is obeyed. Such system could be considered as single compartmental pharmacokinetic model, where the drug concentration drops to zero over time, which is contrary to decaying exponentially with the axis as asymptote. A saw tooth pattern is seen in the concentration time plot for values of the frequency of oscillations, and ratio of the rate constants of infusion and excretion. Bimodal concentration curves cover frequencies of fluctuations [72]. Therefore, understanding of drug-metabolizing enzymes is a key to science of pharmacokinetics that may be used for treatment of drug abuse using enzymotherapy [73]. Zero rate of endogenous substrate production of thymidine and thymine in the present study, indicates that thymidine and thymine are extremely toxic. The finding agrees with the report indicating that, thymidine modulates a number of enzymes in deoxyribonucleic acid (DNA) synthesis or DNA apoptosis. Thymidine in combination with fluorouracil, a metabolite of capecitabine is very useful [40, 74], as a diminished thymidine pool is the mechanism underlying chemoprevention of colon cancer via alpha-difluoromethylornithine [48]. Altered metabolism of thymidine could be caused by abnormal thymidine phosphorylase [41]. Deficiency of dihydropyrimidine dehydrogenase could lead to increased excretion of thymine, uracil, and 5-hydroxymethyl uracil. The affected persons usually become epileptic [46]. Metabolism of oral 5-fluorouracil differs from that of infused form, because the former undergoes more diverse metabolism in the liver and gastrointestinal tract using various enzymes [29]. The metabolites of 5-fluorouracil are dihydrofluorouracil and alpha-fluoro-beta ureidopropionic acid [19]. Dihydrofluorouracil could be detected in 5 min with metabolite of 23.7 μmol in 6o min and half-life of 61.1 min [18]. Therefore, high Km (39.8 mg/L), low enzyme saturation (2.5%), and low rate of endogenous substrate production (0.006/s) in the present study, are suggestive of parenteral fuorouracil administration. Oral 110 mg of tylosin yielded 40% potent metabolites, such as relomycin (tylosin D), desmycosin (tylosin B), dihydrodesmycosin, macrosin (tylosin C) and other 10 metabolites [21], accounting for 61% enzyme saturation and high receptor-binding capacity. Vmax and Km for linear least square using Eadie-Hofstee (193.9,0.0435), Hanes Woolf (216.2,0.0679), Line-Weaver-Burk (195.8; 0.0484), inverse Eadie-Hofstee (215.8;0.0670) and nonlinear least square (212.7; 0.064) for puromicin shows that linear and nonlinear kinetics could fit into Michaelis-Menten order of kinetics [75]. However, the action of potential drugs is based on the inhibition/ activation of oxidoreductase [76]. The rate of ABC transport in NCF- 7 cells obeys Michaelis – Menten kinetics with *V*_*max*_ and km that show similar unimodal distributions, with different maximal cell populations. Higher *V*_*max*_/*km* ratio indicates higher efficiency of transport. Therefore, cell-cycle modulation of multidrug resistant should be taken into account when designing cytotoxic drugs [77]. Signifying that accurate and efficient estimation of enzyme kinetic parameters is beyond Michaelis-Merten equation [9].

### Drugs that obey Michaelis-Menten order of kinetics are liver enzymes inducers and inhibitors

Thiopental and phenytoin are central nervous system depressants that, activate hepatic microsomal enzymes, whereas puromycin inhibits the enzymes. Activation is via oxidation of radicals at carbon 5, N-dealkylation, destruction of barbituric ring and desulfuration of thiobarbiturates [78]. Thiopental increases liver weight, biliary flow and biliary excretion of glutathione conjugate [14]. Phenytoin activates cytochrome P450 and glucuronyl transferase enzymes, hence serum levels of steroids, lamotrigine, tiagabine, vitamin K, cyclosporine, psycotropic, cardiovascular and antineoplastic agents act via CYPIA2, CYP2C9, CYP2C19, CYP3A4 and epoxide hydroxylase. Microsomal enzymes inhibitors, such as metronidazole, mibefradil and isoniazid modulate expression of pegylated – glycoprotein, and, multiple drug resistance proteins 2 and 3 in the gastrointestinal tract [79–81], with attendant consequences of higher cancer mortality, progression of acquired immune deficiency syndrome (AIDS), unwanted pregnancy and rejection of organ transplants. Time course of enzyme induction is governed by receptor up regulation and synthesis of new enzymes. Maximal induction of enzymes is faster with short half-life drugs versus drugs with long half-life. Hence the rate- limiting step is enzyme turn over [82], as could be seen in cases of co-administration of phenytoin with metronidazole. Furthermore, puromycin could be inhibited by chloramphenicol via ribosomes. However, enzymes induction by 3- methylcholanthrene and phenobarbital could be inhibited by puromycin [83]. Isoniazid (30–50 μmol) inhibits activities of enzyme subfamily CYP2CI9 and CYP3A4 [84]. Either thymine or uracil could react with ribonucleosides or deoxyribonucleosides. The ratio of activity between oxyriboside transferring enzymes and deoxyriboside transferring enzyme is 3.5 and reduced to 0.06 in hepatoma [12], but 5-flurouracil forms complex with thymidylate synthase, preventing DNA components of ribonucleic acid (RNA) and DNA [50] in cancer patients. Also 5-fluorouracil inhibits CYP4502C9 [17]. Voriconazole inhibits activities of CYP3A4 in microsomes of liver whereas, metronidazole inhibits CYP2C9 responsible for metabolism of S- warfarin hydroxylation and constitutive androstane receptor that, regulates CYP2C9 and other CYP isozymes [23]. Hence enzyme polymorphism may affect patients’ response to co-administration of the drugs being studied. The most significant enzymes are CYP2D6 and CYP3A4 [85] but CYP3A4 and P-glycoprotein of 0.6 and 0.8 μmol are respectively inhibited [47]. Adverse drug reaction of mibefradil is a reflection of CYP3A4 inhibition in liver and intestine [86]. Induction of hepatic cytochrome (P450) and microoxygenases suggest a mixed type induction by theophylline. Enzymes induction by phenytoin is compensated by increased dose of voriconazole [38]. Inducing and metabolising drugs adversely affect anaesthetics [87]. Liver enzymes cause hepatic hypertrophy [88]. However, substrate depletion and quantification of metabolites can be optimally for determination of kinetic parameters. A typical (non-Michaelis-Menten) occurs when two molecules of the same or different substrates simultaneously activate the active site. The kinetics is biphasic which could be sigmoidal (autoactivation), heteroactivation, substrate inhibition and partial inhibition [72].

### Drug-receptor binding affinity is a function of Michaelis-Menten kinetics

The transport mechanism of *Na*^+^/*k*^+^- ATPase obeys ping-pong mechanism, whereby *Na*^+^ binds to an allosteric non specific site leading to a 2–fold increase in ATPase activity. Michaelis-Menten kinetics is obeyed, when the maximum exponent on the concentration of the varying reactant binds to only one enzyme reaction intermediate. But non–Michaelis–Menten kinetics occurs, when the varying reactant is both substrate and inhibitor (substrate inhibition) or participates in alternative productive pathways or when its stoichiometric coefficient is > 1. Most enzymes follow Michaelis – Menten kinetics [89]. Phenytoin- isoniazid interaction could be managed using pharmacokinetic method of Michaelis – Menten order with *V*_*max*_ in normal range and Km increased by five- fold [49]. Clofazimine and prothionamide may cause drug-drug interaction when co-adminstered with compounds metabolized by CYP 3A4 and CYP2B6, respectively. Whereas isoniazid and rifapentine may cause drug-drug interaction with drugs metabolized by CYP3A4 [67]. Enzyme efficiency is constrained by substrate concentration, genes, and ages, which allows systems modeling from the level of cellular chemical reactions to whole body physiological parameters [90]. For example, Km for carbonic anhydrase is 26 mmol/L [91]. *V*_*max*_ and km estimation by linear methods provide, the most accurate and precise results [92]. Non-compartmental analysis is easier and does not require data modeling, and provides good results as nonlinear mixed effect model for analysis of bioequivalence data, AUC and *C*_*max*_ are estimated by non-compartmental analysis [93]. Mibefradil pharmacokinetics obeys Michaehs-Menten order of kinetics [22], which may be well approximated by a linear model for a single drug exposure, but more than one dose expose nonlineal system, that underestimates the uncertainty in the estimates [3].

### The fundamental pharmacokinetic parameters that could be integrated both in vitro and in vivo

Clearance, volume of distribution, half-life and bioavailability are the fundamental pharmacokinetic parameters [94]. For albumin binding drugs, the effect of albumin on in vivo prediction from in vitro data is very vital for hepatic transporter substrates [95]. High degree of protein binding, narrow therapeutic window, high degree of protein binding drug example phenytoin, and non-linear pharmacokinetics complicate phenytoin dosing. At every high doses R – and S – thiopental exhibit a linear one –compartment model with first order kinetics, and becomes nonlinear one-compartment model with Michaelis-Menten kinetic order. Fraction of unborn R – thiopental used to be higher in this condition [10]. Pharmacokinetics of reversible metabolism, could predict appropriate doses of drug that is subjected to equilibrium in human body [13]. The relation between substrate concentration and dose rate reduces to a linear system [95]. Kinetic constants in the Michelis-Menten metabolism from one enzymatic assay could be approximated using Bayesian computation [96]. Also pharmacokinetics of thymidine, thymine, and fluorouracil is nonlinear in dose [11]. Therefore, terminal half-life is time required to divide plasma concentration (Cp) by two after pseudo-equilibrium has been reached. If the absorption is not a limiting factor, half-life is a hybrid parameter controlled by plasma clearance and extent of distribution. If the absorption is a limiting factor, terminal half-life is a reflection of rate and extent of absorption not elimination [60]. Also lysine 69 is catalytic via *M. tuberculosis* shikimate dehydrogenase and should be used in rational design of antitubercular drugs [97], perhaps with isoniazid. The Hill coefficient of 2.05 ± 0.1 could suggest multiple substrates binding site [98]. Km (1.15 μg/ml) and Vmax (114 μg/h) have been reported for voriconazole administered at 6 mg/kg every 12 h for 24 h and 4 mg/kg at 12 h interval [26], suggesting that pharmacokinetics links efficacy and safety, thereby assist in determination of dosage regimen in clinical practice [99]. Elimination process of two compartment models is by Michaelis-Menten kinetics [100]. In one substrate-one product reversible enzyme reaction, the rapid equilibrium in one direction eliminates rapid equilibrium in the reverse direction. Van Slyke type kinetic constant appears in the rate equation independent of whether steady state, finite time or final equilibrium is attained. Also the reaction could proceed in one direction with fast equilibrium and in the opposite direction with steady-state kinetics. Hence the thermodynamic equilibrium determines that a higher concentration of product or substrate could be reached only with steady-state kinetics [101]. A low Michaelis constant (Km) corresponds to a high binding capacity between enzymes and substrates [102], suggesting that tylosin, puromycin and vorioconazole bind strongly to their receptors as compared to other drugs being studied. In both typical and atypical (non-hyperbolic) parameters, in vitro kinetic parameters are scaled for prediction of in vivo metabolic clearance for dose projection [73].

### Limitations of in vitro-in vivo kinetic translation for optimization of pharmacokinetic/Pharmacodynamic parameters

In vitro-in vivo kinetic translation is useful in drug research and development. However pharmacokinetic parameters such as ADME could be affected at various levels of drug disposition. Phenytoin is postulated to have a limited window of absorption via carrier-mediated mechanism [103]. There was a significant correlation between steady-state serum levels of phenytoin calculated from Vmax and Km values in epileptics. Single doses were initially administered followed by multiple doses [36]. Hence phenytoin could have zero order input and mixed order kinetic ouput in one compartmental model system [104], suggesting that level of plasma phenytoin is not related linearly to dose and change in enzyme activity by co-administered drug, could alter plasma level of phenytoin. What a serious therapeutic setback! More so Km (0.8 μmol/L) and Vmax (1.3 μmol/h) have been reported for phenytoin co-administered with diazepam. The values rose to 50.3 μmol/L and 4.4 μmol/L/h after diazepam elimination [93]. So single intravenous dose, multiple dose and constant dose injection of one compartment models could obey mixed order (Michaelis-Menten) kinetics [55], suggesting that nonlinearity could be observed in drug ADME, which varies from drug to drug, according to route of administration, dosage formulation and diseased conditions [4]. This shows that there is need to establish a dose range with a reasonable relationship between plasma AUC and dosage during subchronic and chronic toxicity studies [105]. The scaling of Vmax and Km gives in vivo metabolic clearance as proven by Vmax/Km ratio [106]. However, many drugs show atypical Michaelis-Menten kinetics that are sigmoidal, biphasic, substrate inhibitory and heterotropic [45], making prediction of in vivo parameters from in vitro data sometimes not exact, and as such should not be based on a single set of data [107]. This is linked to pharmacokinetic/pharmacodynamic modeling that targets drug concentration and effect [108]. Incorrect application of Michaelis-Menten model can result in underestimation of Km and Vmax, but its application to sigmoidal kinetic data could result in overestimation of Km and Vmax at lower concentration of substrate [55], with good correlation between in vitro drug release and in vivo drug absorption, leading to optimized Cmax, Tmax and AUC [109]. Therefore, therapeutic concentration and diagnosis of clinical toxicity of drugs in question, patient compliance and dosage adjustments are clinically useful, especially for patients with greater pharmacokinetic variability [110]. Hence significantly decreased Km than Vmax could result in increased clearance [13], suggesting that linear kinetics removes the regulatory ligand faster, whereas non-linear kinetics delays the removal of regulatory ligand [111]. In spite of the fact that Henri derived equation for enzyme reaction in 1903 [112], significant progress was made by Michelis-Menten in this regard about 100 years ago [113], with many modifications, including the derivation of complex kinetics, where first and second reactions were fast. All these were achieved within the past century [114–118] suggesting that a typical kinetics could be biphasic, homotropic and heterotropic [119]. Hence, some statistical assumptions used in analysis of enzyme kinetic data maybe implicit [120, 121]. Therefore this paper has integrated both in vitro and in vivo equations for identification of enzyme inducing and inhibiting drugs, as well as xenobiotics that undergo enzymatic reactions above Michaelis-Menten order. So what a nice progress made in the area of enzyme kinetics for the past 57 years!

## Conclusion

Many drugs undergoing pharmacokinetics, either obey Michaelis-Menten order (first order, zero order and mixed order) or order above Michaelis-Menten Kinetics. However, the concentrations of drugs and enzymes involved, determine the order of kinetics. More so, in vitro kinetics could be integrated with in vivo Michaelis-Menten kinetics for optimization of pharmacokinetic/pharmacodynamic translation in order to achieve clinical efficacy and safety. Mixed order kinetic drugs are either enzyme inducers or inhibitors.

## Data Availability

The data generated are included in the manuscript.

## Abbreviations

V_o_: Initial velocity
V_max_: Maximum velocity
Cs: Concentration of substrate
Ko: Initial metabolism constant
Co: Initial concentration of substrate
Cl: Clearance
DR: Dose rate
Css: Steady-state concentration
S: Substrate
R: Rate of endogenous substrate production
Clint: Intrinsic clearance
Clu: Urinary excretion
Vd: Volume of distribution
LD: Loading dose
Ki: Inhibition constant
IC_50_: Inhibitory concentration fifty
I_max_: Maximal inhibitory concentration
Ip: Inhibitory potential
Pm: Carrier-mediated permeability
J_max_: Maximal carrier-mediated flux
Co: Initial donor concentration of the substance
MR: Metabolic rate
a: Allometric constant for all mammals
M: Body mass
CMR: Cellular metabolic rate
ds: Change of substrate
dt: Change of time
μ: Kinetic mean
V: Volume of drug
Kcat: Catabolism constant
E_T_: Total enzyme concentration
Es: Enzyme substrate
Kinfus: Infusion constant
ES_ss_: Steady-state-enzyme substrate
A: Lower boundary for concentration
B: Maximum concentration
K: Constant
KE: Elimination constant
KA: Absorption constant
D_1_: First dose
D_2_: Second dose
D_3_: Third dose
β: Elimination rate constant
MRT: Mean residence time
AUC: Area under curve
AUMC: Area under moment curve
T½ *β*: Terminal half = life
Pcl: Plasma clearance
Vc: Volume of central compartment
K_10_: Rate constant of elimination
V_app_: Apparent volume of distribution
Vss: Steady-state volume of distribution
iv: Intravenous
PK: Pharmacokinetics
QT: Depolarization-repolarization of the heart ventricle
CYP: Cytochrome P450
NAD: Nicotinamide adenine dinucleotide
Km: Metabolism constant maximum
DNA: Deoxyribonucleic acid;3-MX: 3-methylxanthine; 1,3-DMU:1,3 dimethyluric acid;1-MU:1-methyluric acid
